# Extracellular vesicles and epigenetic aging clocks in tissue aging: an exosome-focused conceptual framework with a focus on skin

**DOI:** 10.1093/gerona/glag183

**Published:** 2026-07-17

**Authors:** Zeynep Yaren Dinçer, Kaan Zıkşahna, Murat Ihlamur

**Affiliations:** Department of Bioinformatics, Graduate School of Science, Üsküdar University, Istanbul, Turkey; Biruni University Advanced Technology and Research Center (B@MER), Biruni University, Istanbul, Turkey; Department of Basic Medical Sciences, Faculty of Medicine, Biruni University, Istanbul, Turkey; Department of Bioengineering, Faculty of Chemical and Metallurgical Engineering, Yildiz Technical University, Istanbul, Turkey; Biruni University Advanced Technology and Research Center (B@MER), Biruni University, Istanbul, Turkey; Department of Electronics and Automation, Vocational School, Biruni University, Istanbul, Turkey; (Biological Sciences Section)

**Keywords:** Exosomes, Extracellular vesicles, Epigenetic aging clocks, Skin aging, Cellular senescence

## Abstract

Tissue aging is a multifactorial process characterized by cellular senescence, disrupted intercellular communication, and epigenetic alterations, with skin providing a clinically accessible and biologically informative model system. Exosomes and other extracellular vesicles (EVs) are increasingly recognized as mediators of aging-related signaling, whereas DNA methylation–based aging clocks offer quantitative measures of biological age. In this narrative review, we examine the potential interplay between EV/exosome biology and epigenetic aging clocks across tissue-aging contexts, while using skin aging as a primary focus and illustrative model. We review evidence from studies of keratinocytes, dermal fibroblasts, mesenchymal stromal cells, and aging-associated EV cargo, distinguishing studies with direct DNA methylation age readouts from those reporting indirect epigenetic effects. Current evidence indicates that EVs/exosomes from aged or senescent cells may disseminate pro-aging and inflammatory signals, whereas vesicles from young or regenerative sources may support repair and tissue homeostasis. Nevertheless, direct evidence that EVs alter skin epigenetic clock measures remains scarce. In particular, direct experimental evidence demonstrating that aged-cell-derived EVs or exosomes alter DNA methylation clock readings in human keratinocytes or dermal fibroblasts is currently lacking. We propose that skin serves as a useful model for investigating how extracellular signaling interfaces with epigenetic aging dynamics. Therefore, the proposed exosomal–epigenetic clock feedback-loop framework should be interpreted as a hypothesis-generating conceptual model rather than as a validated mechanistic pathway.

## Graphical abstract

**Graphical Abstract:**
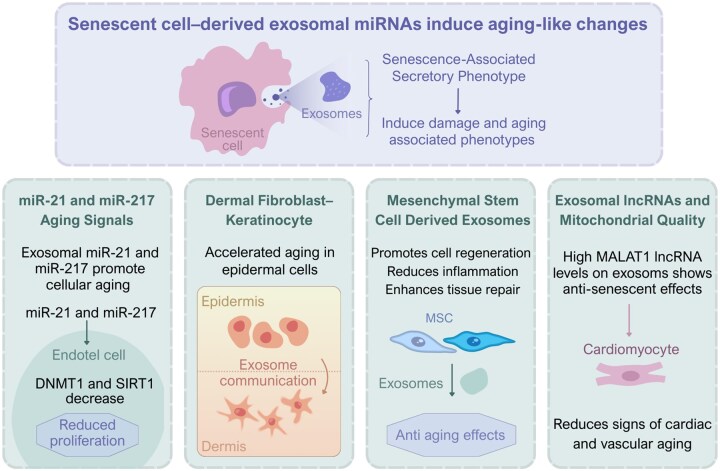

## Introduction

As organisms advance in chronological age, the progressive deterioration of biological functions is defined as aging, a process encompassing changes at cellular, molecular, and systemic levels. Among these, epigenetic alterations are widely regarded as fundamental determinants of aging.^1^^,^^2^ Within the classical hallmarks of aging framework described by López-Otín and colleagues, epigenetic alterations—including disruptions in DNA methylation patterns, dysregulation of histone modifications, and changes in chromatin organization—occupy a central position.^2^ Representative examples of age-associated epigenetic changes include genome-wide fluctuations in DNA methylation levels (regional hypermethylation alongside global hypomethylation), loss of heterochromatin integrity, and reductions in specific histone marks (eg, decreased H3K9 and H3K27 trimethylation).^3^ Such epigenetic alterations contribute to aging-associated phenotypes by reshaping gene expression profiles and, consequently, cellular functions.^4^

One of the most notable advances in the integrative assessment of epigenetic alterations has been the development of epigenetic aging clocks (biological clocks). An epigenetic clock is a mathematical model that estimates an individual’s biological age based on specific DNA methylation patterns.^5^^,^^6^ Among first-generation epigenetic clocks, the model developed by Steve Horvath demonstrated high accuracy in age prediction by leveraging methylation levels at 353 CpG sites derived from multiple tissues.^5^ This pan-tissue epigenetic clock, introduced by Horvath in 2013, was validated across more than 50 healthy human tissues and cell types and exhibited a correlation exceeding 95% with chronological age.^5^^,^^7^ In parallel, Gregory Hannum and colleagues developed an alternative epigenetic clock based on 71 CpG sites, primarily using peripheral blood samples.^7^ Whereas the Horvath clock is broadly applicable across tissues, the Hannum clock predominantly reflects age-related methylation changes in peripheral blood and has been closely associated with immune system aging.^8^ Although both clocks show strong correlations with chronological age (r ≈ 0.97), they also reveal inter-individual variability in aging rates.^5^^,^^7^ When epigenetic age exceeds chronological age, this phenomenon is termed epigenetic age acceleration and elevated age acceleration has been linked to increased mortality and higher risk of age-related diseases in multiple cohort studies.^9^ Horvath has conceptualized the epigenetic clock as an indicator reflecting the cumulative effects of the epigenetic maintenance system.^5^ In this sense, the epigenetic clock may represent the net outcome of accumulated epigenetic damage and repair processes experienced by a cell over time.^10^

At the molecular level, although epigenetic clocks are primarily based on DNA methylation, aging is also accompanied by pronounced alterations in histone modifications and chromatin architecture.^2^ For instance, aging cells typically exhibit a reduction in heterochromatin and transcription-permissive changes within euchromatic regions. Repressive histone marks such as trimethylation of histone H3 at lysine 9 (H3K9me3) are diminished in aged cells, whereas imbalances in histone acetylation levels are frequently observed.^11^ These alterations contribute to compromised genomic stability and the emergence of age-associated gene expression programs, including increased expression of inflammatory genes. In aging cells, sustained activation of DNA damage response pathways, telomere shortening, and metabolic feedback mechanisms are tightly intertwined with epigenetic regulation, collectively shaping the cellular aging phenotype.^12^

Non-coding RNA molecules constitute another important class of epigenetic regulators. Although they do not encode proteins, microRNAs (miRNAs), and long non-coding RNAs (lncRNAs) can modulate gene expression patterns at both post-transcriptional and epigenetic levels.^13^ In particular, lncRNAs have been shown to recruit chromatin-modifying complexes to specific genomic loci, thereby influencing histone modifications and DNA methylation landscapes.^14^^,^^15^ Several lncRNAs and miRNAs associated with aging—such as telomeric repeat-containing RNA (TERRA), which is involved in telomere regulation, as well as miR-34a and miR-21—have been reported to exert either pro-aging or anti-aging effects at the cellular level.^16^ For example, members of the miR-34 family may promote aging by repressing longevity-associated proteins such as sirtuins, whereas certain lncRNAs have been described as playing protective roles in cellular adaptation to stress.^17^ In this review, we therefore focus on the molecular details of DNA methylation clocks as epigenetic markers of aging and examine the potential roles of extracellular vesicles, particularly exosomes, in the epigenetic aging process. Special attention is given to exosomes secreted by skin-resident human cells—keratinocytes, fibroblasts, and mesenchymal stem cells—and to current evidence on how their cargo, including miRNAs, lncRNAs, DNA, and proteins, may modulate epigenetic parameters such as DNA methylation and histone modifications in recipient cells. Because direct skin-specific evidence remains limited, relevant findings from other tissue systems, including cardiovascular, pulmonary, cancer, and systemic aging models, are discussed as supportive but indirect evidence rather than as proof of skin-specific mechanisms. Based on these findings, we propose a hypothesis-generating conceptual model intended to guide future experimental studies.

### Literature search strategy

To inform this narrative review, we searched PubMed/MEDLINE, Scopus, and Web of Science, complemented by targeted Google Scholar queries, for articles published up to 6 January 2026. Search strings combined terms related to extracellular vesicles and exosomes (“extracellular vesicle*”, “EV*”, “small extracellular vesicle*”, “exosome*”), epigenetic aging measures (“epigenetic clock*”, “DNA methylation age,” “DNAmAge,” “epigenetic age acceleration,” “Horvath,” “Hannum,” “GrimAge,” “DunedinPACE”), and skin/aging biology (“skin aging,” “photoaging,” “keratinocyte*”, “dermal fibroblast*”, “senescence,” “SASP,” “inflammaging”). We prioritized primary studies and high-quality reviews relevant to (i) EV/exosome biology in aging or senescence, (ii) mechanisms linking EV cargo to epigenetic regulation, and (iii) applications or model systems pertinent to skin aging. Reference lists of key papers were hand-screened to identify additional relevant publications. Consistent with community recommendations, we use “extracellular vesicles (EVs)” as an umbrella term and reserve “exosomes” for studies that explicitly characterized the small EV fraction with appropriate controls. Where characterization was incomplete, or terminology varied across studies, we preferentially refer to these vesicles as EVs or sEVs in accordance with MISEV recommendations.

## Epigenetic aging clocks: the Horvath and Hannum models

**Figure 1:**
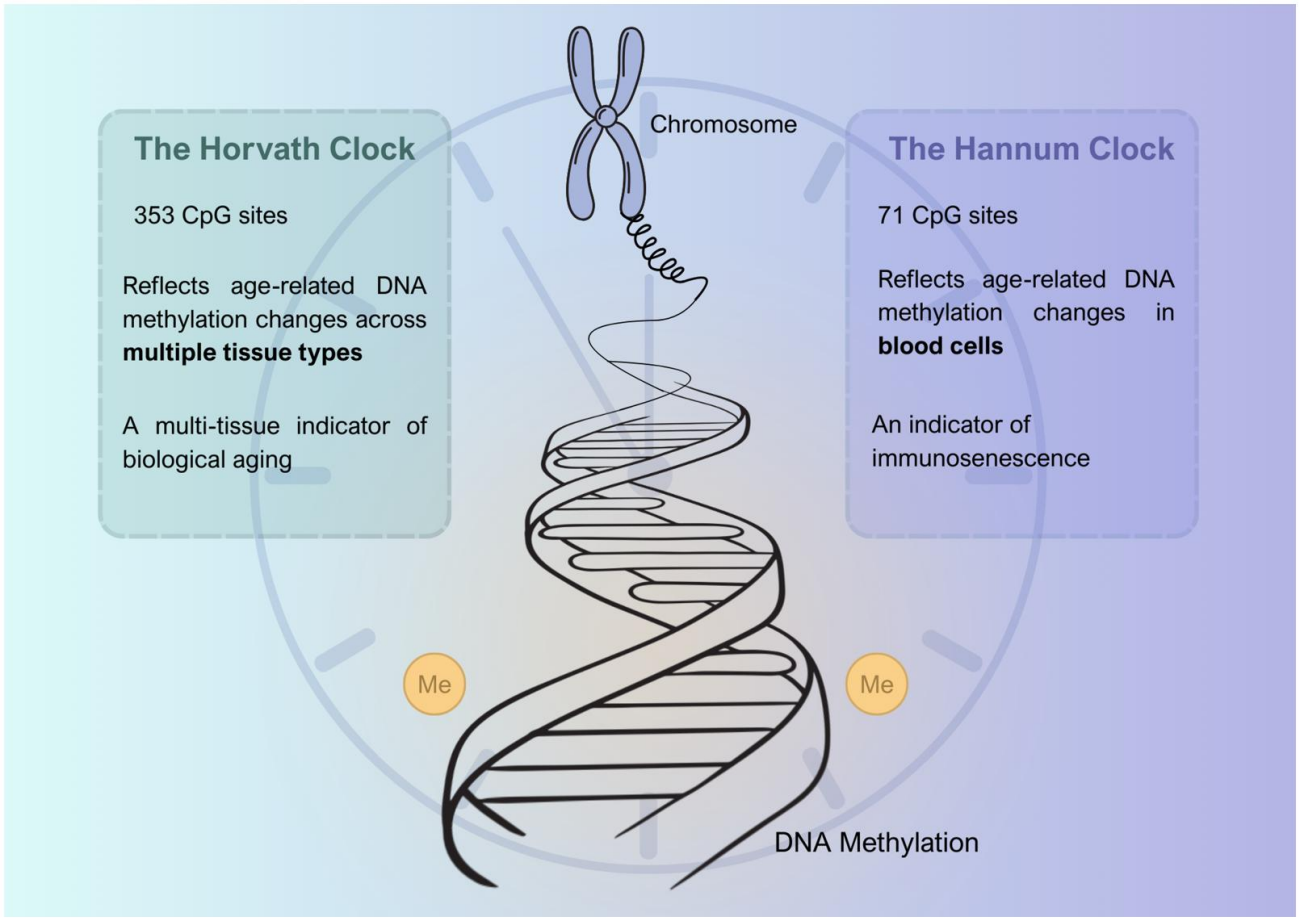
Conceptual comparison of the Horvath and Hannum DNA methylation clocks and their tissue specificity. This schematic highlights the distinction between intrinsic, multi-tissue epigenetic aging captured by the Horvath clock and immune-related, blood-based aging signatures reflected by the Hannum clock.

The concept of the epigenetic aging clock represents a transformative approach in the biology of aging. Whereas traditional aging biomarkers—such as telomere length or cumulative molecular damage—reflect aging indirectly, epigenetic clocks enable the estimation of biological age by directly quantifying chemical modifications across the genome.^12^^,^^18^ Among the first widely adopted epigenetic clock models introduced in 2013 were the Hannum blood-based clock and the Horvath pan-tissue clock, which together established DNA methylation–based age estimation as a robust biomarker framework. The Horvath clock employed a statistical regression algorithm to generate age estimates from tissue-specific methylation profiles. Using the Horvath clock, DNA methylation age was found to be approximately zero in embryonic stem cells and induced pluripotent stem cells, indicating a resetting of epigenetic age in reprogrammed youthful cells. Moreover, the Horvath clock demonstrated progressive age advancement with increasing passage number in cell culture, comparable performance in chimpanzee tissues, and abnormally accelerated aging signatures in cancer tissues.^5^ Collectively, these findings suggested the presence of a conserved, cell-intrinsic epigenetic mechanism underlying aging. Accordingly, the Horvath clock is considered a measure of intrinsic epigenetic age, whereas the Hannum clock more closely reflects immune system aging (Figure 1).

In parallel, Gregory Hannum and colleagues developed an epigenetic clock in 2013 by screening approximately 450,000 CpG sites in human peripheral blood samples and identifying 71 methylation loci most strongly associated with age.^7^ Because the Hannum clock predominantly captures age-related methylation changes in blood cells, it has been widely regarded as an indicator of immune system aging (immunosenescence).^19^^,^^20^ Notably, the Hannum epigenetic age is influenced by the cellular composition of blood, thereby partially reflecting age-associated shifts in immune cell populations.^20^ Accordingly, the extrinsic epigenetic age acceleration index derived from the Hannum clock closely tracks immune aging and exhibits stronger correlations with lifestyle-related factors, such as smoking status and educational attainment.^7^^,^^21^ In contrast, the Horvath clock, which is conserved across tissues and largely independent of blood cell composition, is considered a measure of intrinsic epigenetic age that more directly reflects fundamental, cell-intrinsic aging processes.^5^^,^^20^ Both clock models have demonstrated high accuracy in estimating chronological age across tested populations, typically within an error margin of ±3–5 years.^5^ Nevertheless, substantial inter-individual variability has been observed, with some individuals exhibiting epigenetic ages that deviate markedly from their chronological ages. Individuals whose epigenetic age exceeds their chronological age are often interpreted as undergoing accelerated biological aging, and this phenomenon has been associated with increased risk of multiple age-related diseases.^22^^,^^23^ For example, epigenetic age acceleration has been linked to Alzheimer’s disease, cardiovascular disorders, and declines in physical and cognitive function across numerous studies.^19^^,^^24–26^ Consequently, epigenetic age acceleration derived from epigenetic clocks is now widely accepted as a valuable biomarker in aging research. More recently, second-generation clock models—such as DNAm PhenoAge and GrimAge—have integrated epigenetic age estimates with clinical risk factors, yielding even stronger predictive power for mortality outcomes.^27^^,^^28^ Taken together, these findings suggest that the methylation signatures captured by epigenetic clocks are closely aligned with the underlying biological processes of aging and may provide critical insights into aging mechanisms.^29^^,^^30^

**Figure 2:**
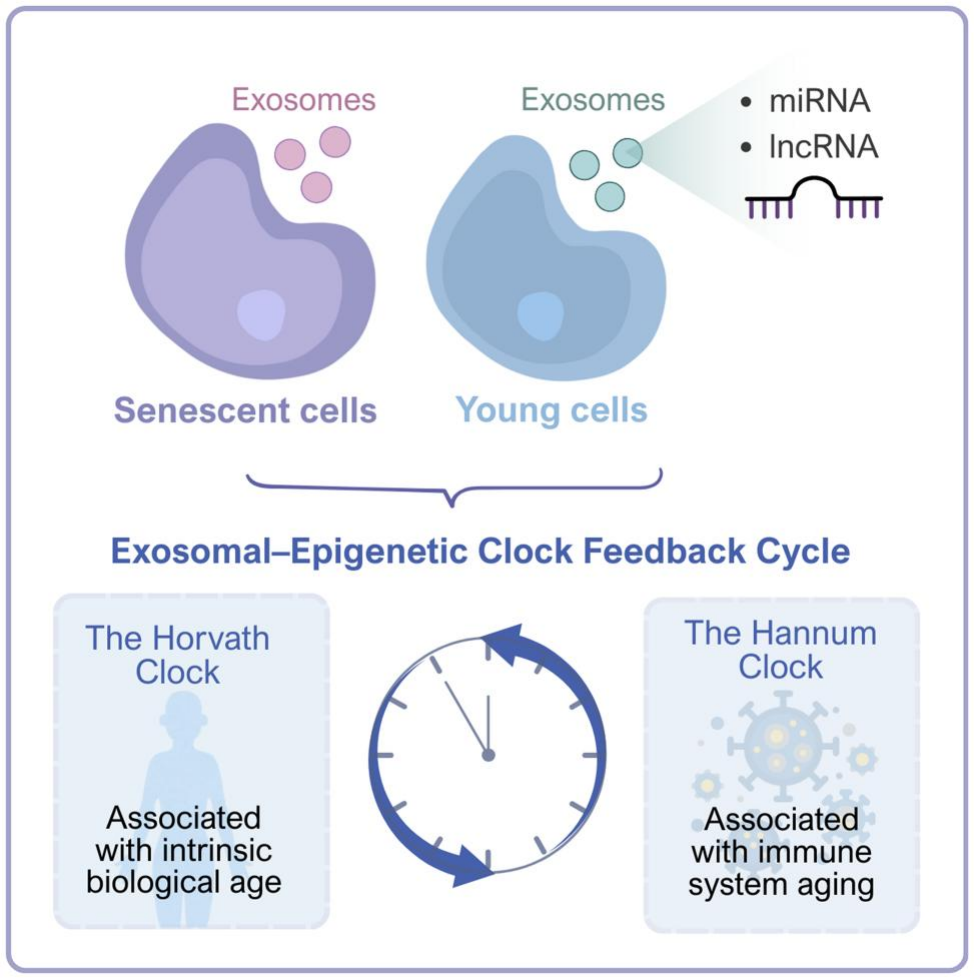
Exosome-mediated modulation of epigenetic aging clocks. Schematic representation illustrating how exosomes released from senescent or young cells carry regulatory non-coding RNAs, such as miRNAs and lncRNAs, that may influence intrinsic (Horvath) and immune-related (Hannum) epigenetic aging pathways in recipient cells, thereby linking intercellular communication with epigenetic clock dynamics.

In his interpretation of what the epigenetic clock measures, Steve Horvath proposed that DNA methylation age reflects the cumulative effect of an epigenetic maintenance or repair system.^5^ This perspective implies that epigenetic regulation is more stringently preserved in young cells, whereas with advancing age this regulatory integrity progressively deteriorates, thereby driving cellular aging. Indeed, many well-established mechanisms associated with aging—such as the accumulation of DNA damage, chronic inflammation, mitochondrial dysfunction, and stem cell exhaustion—are capable of inducing epigenetic alterations. Consequently, the epigenetic clock may represent not only an outcome of aging but also a contributing factor that actively sustains the aging process itself.^31^ Accordingly, recent studies have examined the relationships between epigenetic aging and classical hallmarks of aging. One study reported that epigenetic age acceleration is associated with nutrient-sensing pathways and mitochondrial activity, while remaining largely independent of telomere shortening or the cellular senescence phenotype.^12^ This observation suggests that epigenetic aging occupies a distinct and multidimensional position within aging biology. Although the concept of the epigenetic clock has primarily been developed along the axis of DNA methylation, the underlying mechanisms are likely to encompass a broader spectrum of factors, including histone modifications, chromatin organization, non-coding RNAs, and intercellular signaling processes.^2^^,^^3^^,^^32^ Together, these observations support the view that epigenetic aging clocks not only reflect cell-intrinsic aging processes but may also be influenced by extracellular signals, including exosome-mediated transfer of regulatory non-coding RNAs (Figure 2). Although the Horvath and Hannum clocks remain foundational tools in aging research, future studies specifically designed to evaluate skin aging should preferentially incorporate skin-validated DNA methylation clocks. In particular, the skin and blood clock developed by Horvath and colleagues may provide greater biological relevance for keratinocytes, dermal fibroblasts, and related skin-resident cell populations than general pan-tissue or blood-based clocks alone.

## EVs/exosomes and intercellular communication in aging

Aging is not only characterized by alterations in intracellular processes but also by profound changes in intercellular communication networks.^33^ Altered intercellular communication has been defined as one of the fundamental hallmarks of aging, with chronic low-grade inflammation being a prominent consequence of this disruption.^34^ In aging tissues, certain cell populations secrete deleterious signals into their microenvironment, thereby adversely affecting the function of neighboring cells. In this context, the complex secretory profile released by senescent cells—comprising cytokines, chemokines, growth factors, and proteases—is collectively referred to as the senescence-associated secretory phenotype (SASP).^35^^,^^36^ Beyond recruiting immune cells and sustaining chronic inflammation, SASP components can induce damage and aging-related features in surrounding otherwise healthy cells.^37^ Extracellular vesicles (EVs), identified more recently, constitute a significant component of the SASP.^38^ Throughout this review, we use ‘EVs’ as an umbrella term and reserve ‘exosomes’ for studies that explicitly characterized the small EV fraction consistent with exosomes (eg, size range, enrichment of commonly used markers, and appropriate controls), in line with current community recommendations. Among EV subtypes, small extracellular vesicles (sEVs), often operationally referred to as exosomes in the literature, have attracted particular attention as vesicles typically ranging from approximately 30–150 nm in diameter and secreted by many cell types.^39^ In addition to exosomes and small extracellular vesicles, larger microvesicles released directly from the plasma membrane may also contribute to aging-associated intercellular communication. Several studies suggest that microvesicles can influence inflammatory signaling, mitochondrial stress responses, and tissue homeostasis during aging. However, because the current literature linking microvesicles specifically to epigenetic aging clocks remains limited, the present review primarily focuses on exosome-enriched and small EV populations. Exosomes originate from the fusion of multivesicular bodies with the plasma membrane and serve as vehicles for the transfer of packaged biological information between cells through their diverse molecular cargo.^36^^,^^40^

Exosomal cargo includes proteins, lipids, DNA fragments, messenger RNAs, and various non-coding RNAs, such as microRNAs, long non-coding RNAs, and circular RNAs.^41^ This cargo composition is shaped by the phenotypic state of the originating cell and can influence a wide range of processes in recipient cells, from gene expression to metabolic activity.^42^^,^^43^ Whereas exosomes released from young and healthy cells typically convey signals that support cellular homeostasis, those derived from aged or senescent cells are often enriched in pro-inflammatory mediators and damage-associated signals.^44^^,^^45^ Experimental evidence indicates that senescent cells produce exosomes in greater quantities than proliferative cells and that these vesicles harbor a distinct molecular signature.^45^^,^^46^ For instance, exosomes from aged cells have been reported to contain elevated levels of inflammation-associated proteins, such as cytokines including IL-6, alongside reduced levels of antioxidant enzymes, and to elicit comparable stress responses in recipient cells.^47^^,^^48^ These observations support the notion that EVs—including small EVs often referred to as exosomes—constitute a component of the SASP and may contribute to secondary senescence, whereby senescent traits can spread to neighboring cells via vesicle-mediated signaling.^37^^,^^49^

To elucidate the roles of exosome-mediated cargo in the aging process, microRNAs (miRNAs) have attracted particular attention. miRNAs are small regulatory RNAs that modulate gene expression within cells and, when transferred from one cell to another via exosomes, can function as mobile gene silencers.^50^^,^^51^ Accumulating evidence indicates that exosome-derived miRNAs act as key regulators of age-related diseases and may influence lifespan.^17^ For instance, several well-characterized aging-associated miRNAs, including miR-21, miR-29, and miR-34, can be transported between tissues via exosomes and modulate processes such as tissue regeneration, inflammation, and cellular stress responses in recipient cells.^52–54^ Notably, circulating exosomal miRNA profiles differ markedly between young and older individuals, a divergence that has been suggested to be linked to longevity.^17^ Moreover, specific miRNAs enriched in EVs released from senescent cells have been reported to promote phenotypes consistent with aging-like changes in recipient cells, in part by repressing defined molecular targets.

- miR-21 and miR-217 as aging signals in endothelial cells: In a study conducted in human endothelial cells, replicatively senescent cells were shown to secrete a significantly higher number of exosomes compared with control cells, with these exosomes being markedly enriched in miR-21-5p and miR-217.^55^ Both miR-21 and miR-217 target two key enzymes involved in epigenetic maintenance: DNA methyltransferase 1 (DNMT1) and sirtuin 1 (SIRT1).^56^^,^^57^ SIRT1, a histone deacetylase, exerts anti-aging effects by contributing to genomic stability and telomere maintenance, whereas DNMT1 is responsible for preserving DNA methylation patterns and thereby maintaining epigenetic information across cell divisions.^58^^,^^59^ Exosomes derived from senescent cells and enriched in miR-21/miR-217 were shown to markedly reduce DNMT1 and SIRT1 levels in recipient young endothelial cells, leading to diminished proliferative capacity and the acquisition of a premature aging phenotype. In the same study, exosomal miR-21 levels in healthy human plasma were reported to increase until middle age and subsequently decline in older individuals, forming an inverted U-shaped age trajectory.^55^ This pattern suggests that exosomal miR-21 may serve as a potential biomarker of aging. Collectively, the miR-21 and miR-217 example illustrates how senescent cells may transmit pro-senescent cues to neighboring cells by targeting epigenetic regulatory enzymes through EV/exosome-mediated signaling.
- Dermal fibroblast–keratinocyte communication: In the context of skin aging, exosomal communication between dermal fibroblasts and epidermal keratinocytes is of particular importance. Young fibroblasts can promote epidermal renewal by delivering exosomes containing growth factors and supportive miRNAs to keratinocytes.^60^^,^^61^ In contrast, exosomes secreted by aged fibroblasts may carry signals that impair keratinocyte function. In a 2020 study by Eun-Jeong Choi and colleagues, exosomes released from senescent human dermal fibroblasts were shown to reduce differentiation and compromise barrier function in young keratinocytes. Keratinocytes exposed to these exosomes exhibited slowed proliferation and downregulation of epidermal differentiation markers. In addition, exosomes derived from aged fibroblasts increased the production of inflammatory cytokines, such as IL-6, in young keratinocytes. Together, these findings suggest that EVs/exosomes secreted by aged dermal cells may promote aging-associated functional decline in epidermal keratinocytes, thereby contributing to the intercellular propagation of aging signals within the skin.^61^ Indeed, the presence of senescence markers in both fibroblasts and keratinocytes in aged human skin may reflect the outcome of this deleterious bidirectional communication loop. Conversely, exosomes derived from young fibroblasts have been reported to exert protective effects on aged keratinocytes.^62^^,^^63^ For example, *in vitro* experiments have shown that exosomes obtained from induced pluripotent stem cell (iPSC)-derived young cells enhance proliferation and reverse aging-associated features in UV-damaged aged fibroblasts.^64^ This bidirectional behavior suggests that exosomal communication between dermal and epidermal cells may function both as a driver and a consequence of skin aging.
- Rejuvenating effects of mesenchymal stem cell (MSC)–derived exosomes: Mesenchymal stem cells (MSCs) are key contributors to tissue regeneration in young organisms, and exosomes secreted by these cells have attracted substantial interest due to their anti-aging properties.^65^ A growing body of evidence indicates that MSC-derived exosomes promote cellular renewal, attenuate inflammation, and accelerate repair in damaged tissues.^66^^,^^67^ In particular, the systemic effects of administering exosomes from young MSCs to aged organisms have drawn considerable attention. In a 2024 study by Chen and colleagues, the injection of small extracellular vesicles—predominantly exosomes—isolated from young mice into aged mice significantly extended lifespan and improved physical performance as well as tissue function.^68^ Proteomic analyses revealed that treatment with young exosomes normalized the expression of proteins associated with energy metabolism and mitochondrial function in aged tissues. Mechanistic investigations further demonstrated that specific miRNAs contained within young exosomes increased the expression of peroxisome proliferator-activated receptor gamma coactivator 1-alpha (PGC-1α), thereby stimulating mitochondrial biogenesis and improving energy production in aged cells.^68^ PGC-1α is a critical transcriptional coactivator regulating mitochondrial quality, and its upregulation not only mitigates mitochondrial dysfunction but also shifts cellular energy metabolism toward a more youthful state.^68^^,^^69^ Collectively, these findings provide evidence consistent with the idea that EVs/exosomes from young sources may act as rejuvenating signals in certain experimental models, with reported improvements in functional and molecular readouts suggestive of partial restoration of age-associated decline.
- Exosomal lncRNAs and mitochondrial quality control: Long non-coding RNAs (lncRNAs), another major component of exosomal cargo, also exert significant effects on aging-related processes.^70^ For example, the lncRNA MALAT1 has been implicated as a key regulator in cardiac and vascular aging. Previous studies have shown that MALAT1 is enriched in exosomes derived from young cells and can exert anti-senescent effects in recipient cells.^71^ In a study conducted in 2020 by Xia and colleagues, exosomes released from MSCs under hypoxic conditions were found to carry high levels of MALAT1; these exosomes significantly reduced senescence markers in cardiomyocytes.^72^ Mechanistically, exosomal MALAT1 acts by sponging miR-92a-3p in recipient cells, thereby preventing miR-92a-mediated repression of the autophagy-related gene ATG4a and preserving autophagic flux. Through this mechanism, MALAT1-containing exosomes were shown to prevent doxorubicin-induced premature senescence.^72^ Similarly, other studies have demonstrated that the lncRNA NEAT1, when delivered via MSC-derived exosomes, enhances stress tolerance and delays aging phenotypes in recipient cells.^73^ These examples underscore that exosomes can functionally transfer not only small RNAs but also long non-coding RNAs, thereby reshaping epigenetic and transcriptional programs in target cells.^74^ Beyond RNA species, exosomes may also contain mitochondrial DNA (mtDNA) fragments. This process has been proposed as a mechanism by which cells eliminate damaged mitochondrial components, thereby contributing to mitochondrial quality control.^75^ However, circulating exosomal mtDNA levels have been reported to decline in older individuals compared with younger counterparts, suggesting an age-related reduction in the capacity for mtDNA extrusion.^76^ Consequently, damaged mtDNA may accumulate intracellularly, promoting inflammation, or be released into the circulation as free mtDNA, where it can chronically stimulate the immune system. Because mtDNA resembles bacterial DNA, its extracellular presence can act as a potent inflammatory trigger, a process closely linked to the concept of inflamm-aging.^34^^,^^77^ From this perspective, enhancing exosome-mediated mtDNA clearance may represent a potential strategy to mitigate age-associated inflammation.

These findings indicate that exosome-delivered cargo—including miRNAs, lncRNAs, proteins, mtDNA, and related molecular constituents—can influence multiple dimensions of the aging process.^78^ Exosomes released by aged or senescent cells may disseminate signals that increase the pace of epigenetic clock progression in surrounding cells, whereas exosomes derived from young cells may help restore disrupted cellular homeostasis.^68^^,^^79^ Within this framework, aging emerges as a network-level phenomenon in which intercellular communication loops play a critical role.

Collectively, the studies summarized in Table 1 support the possibility that EV/exosome-mediated communication may influence aging-related pathways and epigenetic regulation in recipient cells. However, direct evidence based on DNA methylation (DNAm) clock readouts remains limited, and the field is further complicated by EV heterogeneity and methodological variability in EV isolation, characterization, and dosing. Therefore, the framework outlined below should be interpreted as a conceptual and hypothesis-generating model rather than a validated mechanistic pathway linking EV biology with epigenetic aging metrics.

**Table 1:** Selected findings linking exosome/extracellular vesicle (EV) cargo involved in intercellular communication during aging with epigenetic axes.

Evidence/topic	Donor source (state)	Recipient target/model	EV/sEV terminology and characterization level	Principal cargo/signature	Epigenetic link (target axis)	Main outcome (summary)	References
**EVs as components of SASP and secondary senescence**	Senescent cells	Neighboring/proliferative cells	EV/exosome	Pro-inflammatory protein/RNA profiles (study-dependent)	Indirect epigenetic reprogramming (inflammation–stress–chromatin responses)	EV-mediated signaling can support secondary senescence; consistent with altered intercellular communication	^37^ ^,^ ^38^ ^,^ ^45^ ^,^ ^47^
**Senescent cell sEVs enhance cancer cell proliferation via EphA2**	Human fibroblasts TIG-3 (replicative/oncogene/DXR-induced senescence) and DXR-induced RPE-1	Cancer cell lines (eg, MCF-7, OVK-18); *in vitro* proliferation assays	Exosome-like small EVs (sEVs)/exosome (small EV fraction isolated from conditioned medium)	EphA2 (upregulated in senescence; enriched in sEVs; increased phosphorylation)	Epigenetic clock not directly measured; findings indicate that senescence-altered intercellular signaling (SASP-EV component) can modulate recipient cell programs (indirect)	Senescent cell–derived sEVs enhance proliferation of certain cancer cells via EphA2–ephrin-A1 interactions; mechanism linked to senescence-associated redox changes and EphA2 loading into sEVs	^46^
**miR-21/miR-217-mediated aging signals in endothelial cells**	Human endothelial cells (replicative senescence)	Young endothelial cells (*in vitro*)	Exosome	miR-21-5p ↑, miR-217 ↑	Targeting DNMT1 and SIRT1 (epigenetic maintenance + deacetylase axis)	DNMT1/SIRT1 ↓; proliferation ↓; premature aging phenotype ↑; plasma miR-21 age-trajectory reported	^56^ ^,^ ^59^
**Dermal fibroblast → keratinocyte communication (young vs aged)**	Dermal fibroblasts (young vs aged)	Keratinocytes (*in vitro*)	Exosome	Homeostasis-supportive vs pro-inflammatory signals (study-dependent)	Indirect epigenetic effects (inflammation/stress → transcriptional programs)	Aged fibroblast-derived EVs impair keratinocyte function; young fibroblast-derived EVs may be supportive	^60^ ^,^ ^61^ ^,^ ^63^
**Aged fibroblast exosomes and impaired epidermal function**	Aged human dermal fibroblasts	Young keratinocytes	Exosome	Pro-inflammatory signals; consistent with increased IL-6	Not an epigenetic clock readout; indirect epigenetic reprogramming inferred from phenotype	Differentiation ↓; barrier function ↓; proliferation slowed; inflammatory responses ↑	^61^
**Repair/regeneration by iPSC-derived “young” EVs**	iPSC-derived young source	UV-damaged/aged fibroblasts (*in vitro*)	Exosome	Cargo consistent with repair/regeneration (study-dependent)	Indirect (DNA damage response, stress–chromatin axis)	Proliferation ↑; aging markers ↓	^64^
**Systemic rejuvenation via young sEV administration**	Young mice (circulating sEVs)	Aged mice (*in vivo*)	sEVs (predominantly exosomes reported)	miRNAs + proteomic changes	Metabolic/mitochondrial axis (PGC-1α pathway) → indirect epigenetic re-setting	Physical performance ↑; tissue function ↑; lifespan ↑; mitochondrial function–related proteins normalized	^68^ ^,^ ^69^
**Anti-senescence/autophagy axis via lncRNA MALAT1**	MSCs (hypoxia)	Cardiomyocytes (model)	Exosome	MALAT1 ↑	MALAT1 → miR-92a-3p sponging → ATG4a/autophagy preservation (indirect)	Doxorubicin-induced senescence ↓; autophagic flux preserved	^70^ ^,^ ^72^
**MIF-preconditioned MSC exosomes reduce cardiac senescence via NEAT1 transfer**	MSCs	Doxorubicin-damaged cardiomyocytes (*in vitro*) and Dox-treated mouse hearts (*in vivo*)	Exosome (50–100 nm; TEM/NTA; CD81 & Flotillin-1 validation)	lncRNA-NEAT1 ↑ (enriched in MIF-exosomes)	NEAT1 → miR-221-3p sponging → relief of Sirt2 repression (Sirt2 ↑); sirtuin (epigenetic deacetylase) axis	Cardiac function (EF/FS) improves; SA-β-gal (+) cells and p16/p27 (± p21) decrease; telomere length and telomerase activity improve; NEAT1 knockdown or miR-221-3p overexpression attenuates effects	^73^
**mtDNA cargo, inflammation, and inflammaging**	Various cell types (age-dependent changes)	Immune system/surrounding tissues	EV/exosome	mtDNA fragments (age-dependent variation)	Innate immune activation → chronic inflammation (indirect epigenetic/IFN programs)	Pro-inflammatory signaling associated with inflammaging; age-related changes in EV-mtDNA levels	^75^ ^,^ ^77^
**Young stem cell sEVs decrease epigenetic age *in vivo***	Young adipose-derived stem cells (young donor state)	Old mice (systemic administration; multi-tissue readouts)	Small extracellular vesicles (sEVs)	Young sEV profile (multi-cargo; not single-factor)	Mouse DNAm clocks (tissue DNAmAge)	sEV treatment improves frailty/healthspan and reduces DNAm-based epigenetic age in old mice	^79^
**ABPC-EVs reverse aging phenotypes and reset blood methylation clocks**	Antler blastema progenitor cells (ABPC; regenerative state)	Aged mice; aged rhesus macaques (systemic)	Extracellular vesicles (EVs)	Regenerative EV cargo signature (proteins/miRNAs; study-defined)	Blood methylation clock analyses (mouse and macaque)	EVs mitigate aging-related phenotypes and reset blood DNAm clock measures, including macaque global epigenetic age signal	^124^
**Human plasma EV proteome correlates with exercise-sensitive DNAm clock**	Human plasma EVs (High-fitness vs Medium/Low-fitness groups)	—(association study)	Plasma EVs (SEC isolation; proteomics)	EV protein cargo (MS-based)	DNAmFitAge (AgeAccelFit); Age/GrimAge accel.	EV protein cargo correlates with AgeAccelFit (but not necessarily other accelerations), supporting human relevance	^125^

Abbreviations: DNAm, DNA methylation; EV, extracellular vesicle; iPSC, induced pluripotent stem cell; lncRNA, long non-coding RNA; miRNA, microRNA; MSC, mesenchymal stem/stromal cell; mtDNA, mitochondrial DNA; SASP, senescence-associated secretory phenotype; sEV, small extracellular vesicle.

## The exosomal–epigenetic clock feedback loop: a hypothetical conceptual model

**Figure 3:**
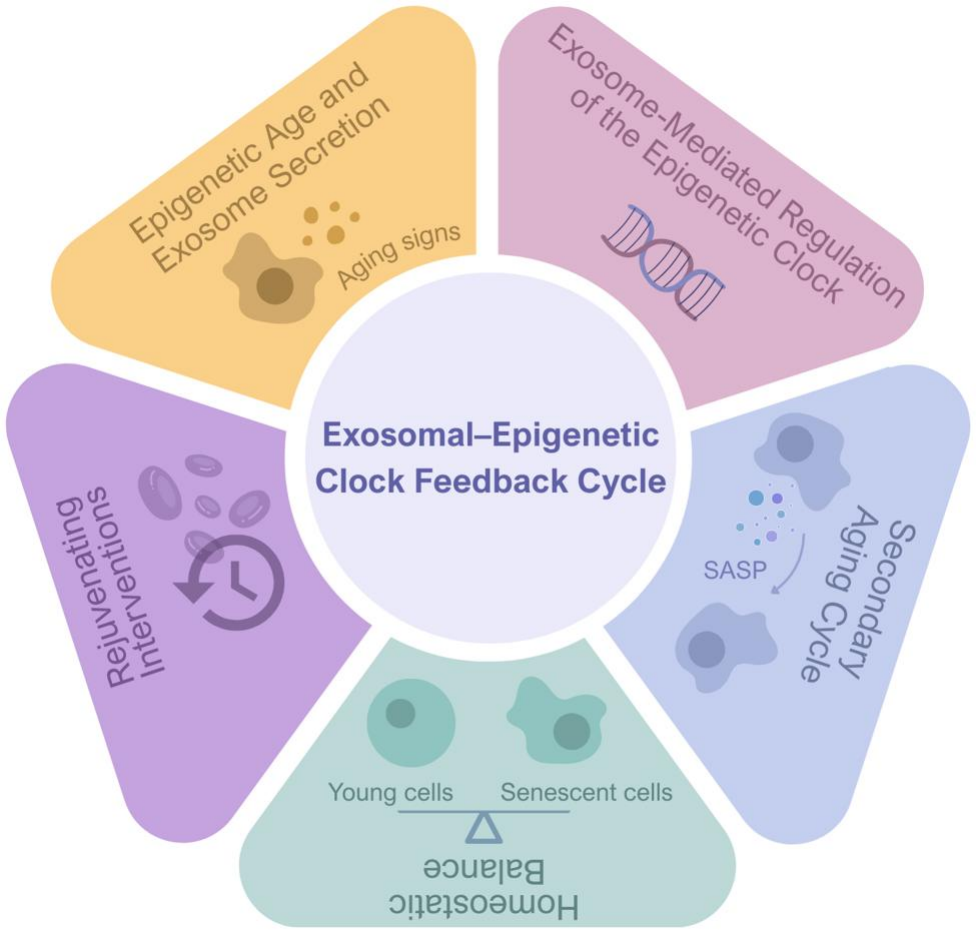
The exosomal–epigenetic clock feedback loop model. The model illustrates a bidirectional interaction in which progression of epigenetic age alters exosomal secretion and cargo composition, while exosome-mediated signaling from aged or senescent cells further modulates epigenetic clock dynamics in recipient cells. This feedback can amplify aging-related processes through senescence-associated signaling or, alternatively, be shifted toward a rejuvenating state by exosomes derived from young cells or through targeted interventions.

Accumulating evidence suggests that aging is shaped not only by damage accumulating within individual cells but also by intercellular signaling processes. Here, we propose the Exosomal–Epigenetic Clock Feedback Loop as a conceptual framework in which cells with advanced epigenetic age display altered EV/exosome signaling, and EV-mediated cues in turn may influence epigenetic clock dynamics in recipient cells, forming a bidirectional interaction that can favor either amplification of pro-aging signals or a shift toward a more rejuvenating state under specific conditions (Figure 3).

### Model-derived, testable predictions

The Exosomal–Epigenetic Clock Feedback Loop framework yields several falsifiable predictions that can be evaluated using DNAm clock readouts alongside EV profiling:

- Within-cell-type coupling: Subpopulations/clones with higher DNAmAge acceleration will secrete EVs enriched for pro-aging signatures (eg, miRNAs targeting DNMT1/SIRT1/HDAC axes and pro-inflammatory/ECM-remodeling proteins), showing a graded relationship between clock state and EV cargo.
- Hypothesized causal directionality: Exposure of young recipient cells to EVs from aged/senescent donors may induce a time- and dose-dependent increase in DNAmAge (or DNAmAge acceleration), accompanied by repression of epigenetic maintenance pathways and increased senescence/DDR signals.
- Propagation across generations: EV passaging (aged EV → young cell → EV produced by the recipient → new recipient) may produce stepwise amplification of pro-senescent phenotypes and DNAmAge acceleration across sequential rounds.
- Loop disruption/rejuvenation: EVs from young or pro-regenerative sources—or blockade of EV biogenesis/uptake—may attenuate or partially reverse DNAm clock progression in aged recipients, with partial restoration of epigenetic maintenance (eg, DNMT1/SIRT1) and reduced SASP/senescence markers.
- Threshold behavior in mosaics: In mixed young/aged (or senescent) cellular mosaics, EV-driven effects on DNAmAge will show non-linear tipping-point dynamics, such that beyond a critical senescent fraction, EV signatures and DNAmAge acceleration increase disproportionately.

### Relationship between epigenetic age and exosome secretion

As a cell’s epigenetic age advances—reflected, for example, by progression of DNA methylation clocks—its gene expression landscape undergoes substantial remodeling.^5^^,^^80^ Aged cells typically exhibit increased production of inflammatory mediators (eg, IL-6, IL-8) and proteases, accompanied by a decline in the expression of repair- and youth-associated factors.^36^ As a downstream consequence of these intracellular changes, the composition of exosomes secreted by the cell also shifts toward a pro-aging profile.^45^^,^^81^ For instance, fibroblasts with advanced epigenetic age have been shown to release a greater number of exosomes with distinct cargo compositions compared with young fibroblasts.^61^^,^^82^ These exosomes may be enriched in miRNAs that suppress epigenetic regulatory enzymes—such as DNMT1, SIRT1, and histone deacetylases (HDACs) (eg, miR-21, miR-217)—or in other molecules that enhance cell-cycle inhibitory pathways.^55^^,^^78^ Similarly, exosomes derived from aged cells may contain increased levels of proteins associated with extracellular matrix degradation and inflammation, including matrix metalloproteinases and cytokines.^47^^,^^83^ Collectively, these observations suggest that cells with an advanced epigenetic clock may generate EV/exosomal signals capable of influencing aging-associated responses in surrounding cells.

### Exosome-mediated modulation of the epigenetic clock in recipient cells

When these exosomes secreted by aged cells are taken up by younger recipient cells, they may perturb the recipient’s epigenetic regulation and potentially contribute to aging-associated changes.^51^ For example, miRNAs delivered via exosomes from aged cells may suppress DNA methylation enzymes such as DNMT1 in recipient cells, disrupting global methylation homeostasis. Such disturbances have been associated with accelerated DNA methylation–based epigenetic aging.^84^^,^^85^ In addition, exosome-derived signals can activate DNA damage response pathways, promote loss of heterochromatin, or induce senescence-associated gene expression programs in recipient cells.^86^ A representative example of this cross-talk is provided by exosomes released from aged dermal fibroblasts, which have been shown to impair proliferation in epidermal keratinocytes.^87^ Through these mechanisms, the advanced aging state of one cell could plausibly influence aging-associated molecular programs in neighboring cells via EV/exosome-mediated signaling. However, whether such signaling is sufficient to produce measurable and durable shifts in DNAm clock readouts remains an open experimental question. Therefore, this process should be viewed as a testable mechanism of local aging-associated signal propagation rather than as an established mechanism that synchronizes epigenetic aging across tissues.

### Secondary aging signals and constrained feedback-like dynamics

If a recipient cell enters an epigenetically accelerated aging trajectory as a consequence of deleterious exosomal signals, it may progressively acquire a senescent-like cellular phenotype itself.^88^ As a result, this cell begins to exhibit a SASP-like secretory profile and to produce its own exosomes enriched with pro-aging cargo. This process may enable the transmission of aging-associated signals from cell to cell, forming a constrained feedback-like interaction that could amplify aging-related signaling only under permissive conditions.^89^ Notably, a process initiated by senescence in a limited number of cells within a tissue microenvironment can, over time, propagate to broader cell populations in a wave-like manner. For example, during the development of pulmonary fibrosis, exosomes released from a small number of damaged cells can activate fibroblasts, promoting their senescence and fibrotic transformation, ultimately leading to widespread tissue stiffening.^90^ Through such mechanisms, exosome-mediated secondary senescence loops may critically influence the overall rate at which aging progresses at the organismal level.

### Rejuvenating interventions and disruption of the feedback loop

A key implication of this model is that the proposed feedback loop may be disrupted by external interventions. Administration of exosomes derived from young sources to aged tissues, or inhibition of the production or uptake of deleterious exosomes, may attenuate or, in selected experimental contexts, partially reverse specific aging-associated signaling changes.^88^ One mechanistic explanation for classical experiments demonstrating rejuvenation of aged mice by young blood plasma is that exosomes present in young plasma reactivate beneficial gene expression programs in aged cells.^59^ In a 2025 study, Bi and colleagues reported that administration of exosomes derived from human embryonic stem cells to aged mice extended lifespan and induced molecular markers of rejuvenation across multiple tissues.^91^ These effects were associated with the high abundance of youth-associated factors, such as miR-302, within the exosomal cargo, which were shown to reinitiate cell-cycle activity in aged cells. Specifically, miR-302b suppresses cell-cycle inhibitors including p21 (Cdkn1a) and Ccng2, thereby reactivating previously arrested cell-cycle programs in aged cells and enabling a subset of these cells to reenter proliferation.^91^ This study provides evidence that selected exosomal cargo can modulate aging-associated cellular phenotypes in experimental settings, supporting the possibility that components of the proposed model may be experimentally tractable. According to this framework, exosome-based interventions with pro-regenerative properties may shift the local signaling environment away from pro-aging amplification and toward a more homeostatic or repair-supportive state. For example, young exosomes may elevate SIRT1 levels and stabilize DNMT1 in aged cells, thereby slowing epigenetic clock progression. In parallel, improvements in mitochondrial function and reductions in inflammatory signaling may further contribute to partial modulation or restoration of selected aging-associated cellular phenotypes.^92^^,^^93^

### A balanced homeostatic state

In complex organisms, tissues exhibit a mosaic composition in which young and aged cells coexist. The proposed feedback-loop model posits that intercellular interactions within this mosaic critically shape the trajectory of aging. In young organisms, exosomal communication is maintained in a balanced state that preserves tissue integrity, limits damage propagation, and supports regeneration.^94^^,^^95^ As aging progresses, the accumulation of cellular stress and damage accelerates the transition of certain cells toward an aged state at epigenetic layers such as DNA methylation and chromatin organization. This transition is accompanied by an increased burden of senescent cells. Beyond the canonical components of the SASP, senescence-associated extracellular vesicle/exosome cargo further amplifies pro-inflammatory and pro-senescent paracrine effects within the tissue microenvironment, thereby disrupting homeostatic balance and promoting the dominance of pro-aging signals.^96^ Once a critical threshold is exceeded—when senescent cell burden and inflammation reach sufficient levels—the SASP and exosome/EV-mediated signals from aged cells may contribute to a positive feedback-like process that increases stress and senescence in neighboring cells. Consequently, cellular phenotypes shift toward an aged state, tissue homeostasis deteriorates, and the rate of organismal aging accelerates alongside a loss of functional reserve. Importantly, this model does not imply an unrestricted or runaway aging process. *In vivo* tissues contain multiple buffering mechanisms that may limit the spread of EV/sEV-mediated aging-associated signals, including immune-mediated clearance of senescent cells, DNA repair responses, antioxidant and proteostatic defence systems, extracellular matrix remodeling, dilution by functionally competent neighboring cells, and senolytic or senomorphic activities. Therefore, the proposed feedback-like process should be interpreted as threshold-dependent and context-specific. Under low senescent-cell burden or efficient tissue surveillance, EV/sEV-mediated signals may remain buffered and insufficient to shift tissue-level aging trajectories. In contrast, when senescent-cell accumulation, inflammation, tissue damage, or impaired immune clearance exceed local homeostatic capacity, EV/sEV-mediated communication may contribute to non-linear amplification of aging-associated phenotypes.^97^^,^^98^

Experimental validation of this theoretical framework represents an important objective for future aging research. In particular, mapping the relationship between single-cell epigenetic age and the composition of exosomes secreted by the same cells would help substantiate key components of the model. In addition, administering exosomes isolated from young or aged cells to distinct recipient cell populations and monitoring resulting changes in epigenetic clock readouts would provide direct tests of causality. Such experimental approaches may also clarify the efficacy of exosome-based interventions aimed at modulating aging trajectories.

## Discussion

This review examines the effects of exosomes on epigenetic aging and offers a perspective informed by evidence from human-derived data and animal models. The proposed *Exosomal–Epigenetic Clock Feedback Loop* model conceptualizes aging as a process shaped not only by cell-intrinsic dynamics but also by environmental cues and intercellular interactions. This view implies that strategies aimed at modulating aging-associated phenotypes may need to move beyond targeting single cells in isolation and instead address communication networks operating at the tissue and organ levels.

At the same time, the current evidence should be interpreted cautiously. Many of the studies supporting EV/exosome-mediated effects in aging demonstrate changes in senescence markers, inflammatory mediators, mitochondrial function, proliferation, or tissue repair capacity rather than direct alterations in DNA methylation age. Moreover, epigenetic clocks are best regarded, at present, as quantitative biomarkers that integrate aspects of biological aging, rather than as proven causal mediators of the aging process. Accordingly, concordance between EV-induced phenotypic changes and epigenetic clock shifts cannot be assumed without direct experimental validation. This distinction is important because some EV-mediated responses may reflect adaptive stress signaling or transient changes in cell state rather than durable acceleration or reversal of epigenetic aging.

Evidence in the literature supports the notion that cellular aging can display collective behavior.^1^^,^^3^^,^^5^ For example, the selective clearance of senescent cells (senolytic therapies) has been reported to extend lifespan and improve tissue function in multiple mouse studies, an effect that has been attributed in part to the removal of SASP/exosome-mediated signals propagated by aged cells.^79^^,^^91^^,^^99^ Similarly, upon the application of rejuvenating interventions—such as partial reprogramming via Yamanaka factors or young plasma transfer—the conversion of a subset of cells toward a youthful phenotype can also improve the function of neighboring cells.^100^^,^^101^ This phenomenon is consistent with, but does not by itself prove, the type of compensatory or negative feedback-like dynamics proposed in the present conceptual model. Notably, exosomal cargo composition and its functional consequences in recipient cells are context-dependent. For instance, exosomes released by cancer cells can disseminate both aging-like alterations and tumor-promoting effects to the surrounding microenvironment, whereas exosomes derived from young stem cells may stimulate tissue renewal.^102^^,^^103^ It is therefore important to recognize that exosomes are not invariably detrimental during aging and may also serve compensatory or adaptive roles.^97^ For example, the removal of damaged mitochondrial components via exosomes may function as a short-term protective mechanism. However, as this process becomes insufficient with advanced age, it may ultimately contribute to inflammation.^104^^,^^105^ Accordingly, future therapeutic approaches targeting exosome modulation will likely require careful calibration. In this context, the question of whether epigenetic clocks can be slowed by enriching or depleting specific miRNAs or lncRNAs within exosomal cargo becomes particularly salient.^106^^,^^107^ For example, exogenous supplementation of youth-associated factors such as miR-221—reported to decline in exosomes from aged cells—has been shown to exert rejuvenating effects in the cardiovascular system.^108^ Likewise, delivery of lncRNAs such as MALAT1 or H19 via therapeutic exosomes may enhance the regenerative capacity of aged cells.^109^^,^^110^

Advances in technology are increasingly enabling more precise control over exosome biogenesis and cargo loading processes, supporting the growing investigation of exosome-based interventions as a potential therapeutic strategy.^111^ In particular, skin aging represents an ideal platform for the development of exosome-based cosmetic or therapeutic products.^112^ Several clinical studies have reported that topical or injectable administration of mesenchymal stem cell–derived exosomes enhances collagen production in the skin and reduces wrinkle formation.^113–115^ Nevertheless, issues of standardization and safety remain critical. Exosomes may elicit immune responses or exert off-target effects, underscoring the need for rigorous quality control and risk assessment in clinical applications. Accordingly, EV-based anti-aging interventions should still be considered investigational and should not be interpreted as ready for routine clinical application in skin aging without rigorous safety, dosing, biodistribution, manufacturing, and long-term outcome studies.

Another important consideration is the potential use of epigenetic clock measurements to evaluate the efficacy of anti-aging interventions. If the exosomal feedback-loop hypothesis holds true, successful rejuvenating treatments should be accompanied by a deceleration or partial reversal of epigenetic aging clocks. Indeed, in the recent small-scale, uncontrolled TRIIM trial, a treatment regimen combining growth hormone, dehydroepiandrosterone (DHEA), and metformin was associated with a modest reversal of DNA methylation–based epigenetic age relative to baseline. However, the generalizability of these findings is limited by the study design and sample size.^116^ Although the underlying mechanisms in this trial were not explicitly attributed to exosomes, the study is nonetheless notable for demonstrating that epigenetic age is a modifiable parameter. Looking ahead, epigenetic clock readouts could offer an objective metric for evaluating interventions such as exosomes derived from young donors, for example by quantifying the degree to which epigenetic age is reduced after treatment. The influence of exosomes on epigenetic aging lies at a rapidly evolving, multidisciplinary intersection of aging biology. The model and literature discussed in this review collectively indicate that intracellular and extracellular processes form an integrated aging network. Within this network, EVs may act as important mediators of intercellular communication that could influence aging-associated trajectories while also representing potential targets for future investigation.

## Limitations, challenges, and future directions

### Limitations of the current evidence base

Despite growing interest in EVs, including exosomes, as modulators of aging biology, the causal linkage between EV signaling and quantitative shifts in epigenetic aging measures (eg, DNAm-based clocks) remains insufficiently established. A large fraction of the literature demonstrates EV effects on phenotypes frequently associated with aging (senescence-associated inflammation, mitochondrial dysfunction, impaired repair capacity), yet epigenetic clock outputs are seldom incorporated as prespecified endpoints, limiting the ability to infer whether EV perturbations truly alter the pace of epigenetic aging or primarily influence downstream correlates.^22^^,^^117^

A second limitation concerns model heterogeneity. Evidence is drawn from diverse *in vitro* stress paradigms, animal studies, and a comparatively small set of human observations. These contexts vary in baseline epigenetic trajectories, cell-type composition, exposure histories (notably UV in skin), and immune clearance, which can substantially modulate both EV biogenesis and recipient-cell responsiveness. Consequently, generalization across tissues, species, and exposure contexts remains uncertain, and apparent agreement between studies may partially reflect shared stress responses rather than convergent aging mechanisms.^118^^,^^119^

Third, interpretation is constrained by measurement and technical variability on both sides of the proposed EV–epigenetic interface. DNAm clocks differ in training data, tissue specificity, susceptibility to cell-composition shifts, and sensitivity to batch effects.^18^^,^^120^ In parallel, EV preparations can differ markedly in vesicle subtype content and co-isolated material, and methodological choices (isolation, storage, quantification, and normalization) may drive variability comparable to biological effects. These factors complicate cross-study comparisons and impede robust meta-analytic synthesis.^121^^,^^122^

Finally, mechanistic depth is uneven. Current discussions disproportionately emphasize readily profiled cargos (eg, miRNAs) and canonical pathways (inflammation, mitochondrial stress), whereas potential EV influences on chromatin organization, histone modifications, and nuclear architecture—and their relationship to clock CpGs—remain relatively underexplored. The field therefore risks conflating association, mediation, and causation without appropriately designed perturbation studies.^18^^,^^119^

### Methodological and translational challenges

- Standardization and reporting. Terminology and characterization standards are not uniformly applied, especially when exosome is used to describe small EV fractions without comprehensive validation. Greater adherence to community guidelines and transparent reporting (isolation workflow, contaminant assessment, marker panels, particle/protein metrics, and functional controls) is necessary to improve reproducibility.^122^
- Dose definition, normalization, and potency. Functional comparability is challenging because EV output and cargo loading change with donor state (aging, senescence, and stress). Normalizing by particle number or protein mass does not guarantee biological equivalence. The field would benefit from harmonized dosing frameworks and potency assays linked to mechanistic readouts.^123^
- Biodistribution, specificity, and safety considerations. For translational applications, uncertainties persist regarding EV biodistribution, cellular uptake specificity, persistence, and potential off-target effects. In particular, interventions that aim to enhance repair or regeneration should be evaluated for context-dependent trade-offs, including pro-fibrotic, pro-inflammatory, or pro-proliferative signaling in susceptible settings.^78^
- Human evidence and confounding. Human studies frequently remain cross-sectional and are vulnerable to confounding (comorbidities, medications, smoking, UV exposure history, and environmental factors). Longitudinal designs with repeated sampling and careful covariate handling are required to determine whether EV signatures precede, accompany, or follow epigenetic aging changes.^118^
- Clock selection and tissue relevance. Epigenetic clocks are not interchangeable, and clock choice should be aligned with the sample type (blood vs skin tissue vs cultured cells), the biological question (chronological-age tracking vs morbidity/mortality risk vs pace-of-aging), and the expected effect size and durability of EV perturbations. DNAm clocks differ in training data, tissue specificity, susceptibility to cell-composition shifts, and sensitivity to batch effects; therefore, conclusions about rejuvenation or acceleration require careful preprocessing and principled clock selection. For skin-focused EV studies, tissue-appropriate clocks should be prioritized. Where feasible, clocks developed or validated for skin and blood or for relevant epithelial/mesenchymal contexts (eg, fibroblast- and keratinocyte-relevant applications) can reduce misinterpretation due to cross-tissue transferability limitations (Horvath et al., 2018). For blood-based human studies, it is often informative to report at least one risk- or phenotype-linked clock (eg, PhenoAge/GrimAge) and, when available, a pace-of-aging measure (eg, DunedinPACE), recognizing that immune-cell composition and inflammatory states can strongly influence blood-derived readouts. Practical reporting recommendations for EV–clock studies include: (i) reporting both raw DNAmAge and age-acceleration metrics (revisualized for chronological age where appropriate), (ii) explicitly stating the tissue/cell type, passage number (for cultured cells), and cell-composition handling (deconvolution or controlled cell mixtures), (iii) describing DNAm preprocessing/QC and batch correction, and (iv) interpreting short-term shifts cautiously unless supported by time-series and washout/durability assessments.^118^^,^^120^

### Future directions

- Causal perturbation studies with clock-based endpoints. A priority is rigorous testing of whether EVs can shift epigenetic aging readouts under controlled conditions. This requires time-series designs that combine (a) defined donor states (young/aged/senescent; intrinsic vs extrinsic stress), (b) stringent EV characterization, (c) standardized dosing and uptake controls, and (d) preplanned DNAm clock and chromatin/transcriptomic endpoints in recipient cells.
- Cell-type–resolved mapping in physiologically relevant skin systems. Skin aging is shaped by multi-cellular interactions; therefore, organotypic or microphysiological models that incorporate keratinocytes, fibroblasts, and immune components—under defined UV and inflammatory exposures—will be important to distinguish cell-intrinsic from microenvironment-driven EV effects. Integrating single-cell or deconvolution strategies could clarify which compartments contribute most to EV production and clock dynamics.
- Multi-omics integration and EV aging signatures. Combining EV cargo multi-omics with DNAm clocks and functional phenotypes may enable the construction of EV-derived aging signatures and identification of cargos that most consistently track with biological aging rather than disease-specific changes. Importantly, such models should be validated across cohorts and platforms.
- Mechanistic expansion beyond DNA methylation alone. To connect EV signaling to epigenetic aging more mechanistically, future work should interrogate chromatin accessibility, histone marks, and 3D genome organization alongside DNAm changes. This may reveal whether EVs act on clock CpGs directly or reshape upstream chromatin states that secondarily influence methylation maintenance.
- Translational development with conservative claims and robust safety frameworks. When considering EV-based interventions (including dermatologic applications), emphasis should be placed on manufacturing standardization, release criteria, potency assays, and long-term safety monitoring. Claims should remain conservative unless supported by durable effects across multiple molecular and functional endpoints and a favorable risk profile. In many contexts, it may be more appropriate to frame outcomes as modulation of age-associated pathways or improvement of specific aging-related readouts, rather than implying broad reversal of aging.
- Context dependence and thresholds. EV signaling may be adaptive in acute stress yet maladaptive under chronic exposure. Identifying context-dependent thresholds—when EV-mediated communication transitions from compensatory to propagating dysfunction—could help define actionable intervention windows and refine therapeutic targets.

## Conclusion

The concept of epigenetic clocks provides a powerful tool for capturing the molecular imprint of aging within DNA methylation patterns and is increasingly regarded as a gold-standard approach for estimating biological age. In this review, we comprehensively addressed the core mechanisms underlying epigenetic clocks and examined, in particular, how these clocks may be modulated via exosome-mediated intercellular communication. Current evidence indicates that EVs or exosome-enriched preparations secreted by aged cells may promote aging-associated phenotypes in younger cells; conversely, vesicles derived from young or regenerative sources may support partial restoration of selected youthful cellular features in experimental models. This bidirectional interaction was articulated and discussed through the theoretical framework we term the *Exosomal–Epigenetic Clock Feedback Loop*. The model emphasizes that some aging-associated cellular phenotypes may be influenced by intercellular communication and, under defined experimental conditions, may show partial modulation by EV/exosome-mediated signals.

This perspective is relevant to both basic and clinical sciences. Understanding the role of intercellular communication networks in aging may lay the groundwork for developing holistic anti-aging strategies. For example, correcting age-disrupted exosomal cargo profiles or therapeutically administering exosomes from young sources could potentially improve organ function. Early translational and clinical investigations of EV/exosome-based interventions have begun to emerge in areas such as skin rejuvenation and wound repair, although their interpretation remains limited by heterogeneity in product characterization, dosing, endpoints, and follow-up duration. Epigenetic clock measurements may provide one useful exploratory readout for evaluating such interventions, provided that tissue specificity, clock selection, and study design limitations are carefully considered.

Overall, the potential interplay between EV/exosome-mediated communication and epigenetic aging may offer a useful conceptual perspective for studying aging processes. Although research in this area remains at an early stage, the available findings are promising. The notion that certain aging-associated phenotypes may be modifiable through interventions targeting cellular communication networks is gaining increasing attention. We anticipate that the theoretical framework presented here will help guide future efforts to develop anti-aging therapies and strategies for healthy aging.

## Data Availability

No new data were generated or analyzed in support of this research. Data sharing is not applicable.
